# Stigmatization and the Experience of Informal Dementia Caregivers in Nigeria

**DOI:** 10.1093/geroni/igaa057.887

**Published:** 2020-12-16

**Authors:** Candidus Nwakasi, Kate de Medeiros, Darlingtina Esiaka

**Affiliations:** 1 University of Southern Indiana, Evansville, Indiana, United States; 2 Miami University, Oxford, Ohio, United States; 3 Union College, Union College, New York, United States

## Abstract

There is no formal word for dementia in Nigeria. Instead, some Nigerians, in their effort to make sense of dementia symptoms, use descriptions that may result in stigmatization of people living with dementia and their families. With Nigeria’s rapid aging, increased risk of dementia, and lack of formal long-term care, this study focused on the impact of stigma on the caregiving experiences of Nigerian women. This exploration is significant as adult females in Nigeria are the pillar of informal caregiving in the country. The study employed a qualitative descriptive method. Semi-structured interviews were conducted with a purposive sample of 12 adult informal female caregivers in Anambra, Nigeria. Data were then transcribed, coded and analyzed for themes. Afterwards, focus groups of 21 adult Nigerians residing in Ohio, US, were conducted to offer more contextual insight on the findings. The three major themes identified were: 1) negative views of dementia symptoms (e.g., witchcraft, madness), 2) caregiving protects against stigmatization (e.g., by keeping family members out of sight), and 3) stigma and caregiving support such as adult children abandoning parents with dementia because of the stigma associated with dementia. Given the overwhelming presence of stigma in all aspects of dementia to include dementia caregiving, results point to the critical need for better strategies to help strengthen informal caregiving in Nigeria. This includes culturally appropriate dementia education for families and caregivers, and formal long-term care policies that include care support in a rapidly aging Nigeria.

